# Synthesis and Properties of Polyamide 6 Random Copolymers Containing an Aromatic Imide Structure

**DOI:** 10.3390/polym15132812

**Published:** 2023-06-25

**Authors:** Yingwei Zhang, Chunhua Wang, Yong Yi, Wenzhi Wang, Jun Yang

**Affiliations:** 1 National and Local Joint Engineering Research Center of Advanced Packaging Material Research and Development Technology, School of Packaging and Materials Engineering, Hunan University of Technology, Zhuzhou 412007, China; zhangyingwei@stu.hut.edu.cn (Y.Z.); yiyong@stu.hut.edu.cn (Y.Y.); wangwenzhi@hut.edu.cn (W.W.); 2 Zhuzhou Times New Material Technology Co., Ltd., Zhuzhou 412007, China

**Keywords:** polyamide 6, poly(amide imide), random copolymerization

## Abstract

In order to adjust the properties of polyamide 6 (PA6) and expand its application, a new strategy of introducing an aromatic imide structure into the PA6 chain through the random copolymerization method is reported. The diimide diacid monomer was first synthesized by the dehydration and cyclization of pyromellitic dianhydride and 6-aminocaproic acid before it reacted with 1,6-hexamethylene diamine to form poly(amide imide) (PAI) salt, and finally synthesized PA6/PAI random copolymers containing an aromatic imide structure by the random copolymerization of ε-caprolactam and PAI salt. The introduction of an aromatic imide structural unit into the PA6 chain could have a great influence on its properties. As the content of PAI increases, the crystallinity (*X*_c_) and melting temperature (*T*_m_) of the PA6/PAI random copolymer gradually decrease, but its glass transition temperature (*T*_g_) increases obviously. When the PAI content is 20 wt%, the copolymer PA6/PAI-20 has the best comprehensive performance and not only has high thermal stabilities but also excellent mechanical properties (high strength, high modulus, and good toughness) and dielectric properties (low dielectric constant and dielectric loss). Moreover, these properties are significantly superior to those of PA6. Such high-performance PA6 random copolymers can provide great promise for the wider applications of PA6 materials.

## 1. Introduction

Polyamide 6 (PA6) as an excellent engineering plastic that has been widely investigated and applied in automotive, electrical, packaging, and other industrial fields due to its lightweight, good mechanical properties, excellent chemical resistance, outstanding processability, and low cost [1,2,3,4,5]. With the rising demand for products in diversified markets, the single pure PA6 resin cannot meet all the requirements. In order to adjust its properties and expand its applications, the development of new PA6-modified varieties has become the focus of current research.

Copolymerization is a common modification method in polymer chemistry, and the properties of copolymers can be adjusted according to the need to change the types of comonomers and their relative proportions [6,7,8,9,10]. In order to adjust the properties of PA6, it is necessary to design and synthesize new copolymers by introducing some rigid structures [11]. Aromatic polyimide (PI) is well-known as a high-performance polymer material with excellent mechanical properties and high thermal stability due to its highly rigid chain structure and strong chain interaction [12,13,14,15,16]. Therefore, introducing an aromatic imide structure into the PA6 chain is an effective modification method. Several examples in the literature have reported the linkage of the aliphatic PA6 chain with a rigid aromatic imide structure by block and graft copolymerization [17,18,19,20,21]. For example, PA6-b-PI-b-PA6 copolymers were synthesized using polyimide oligomers that were end-capped with phenyl 4-aminobenzoate to activate the anionic polymerization of ε-caprolactam, and PA6 chains grew from both ends of the polyimide oligomers to form triblock copolymers [19]. All the PA6-b-PI-b-PA6 copolymers had higher tensile moduli and tensile strengths than PA6, but their elongations at break were much lower than PA6. PI-g-PA6 copolymers were synthesized by the polymerization of phenyl 3,5-diaminobenzoate with several diamines and dianhydrides, and the polyimide oligomers containing pendant phenyl ester groups were then used as activators for the anionic polymerization of ε-caprolactam [20]. These block and graft copolymers exhibited good melt processability, and their thermal stability, impact strength, and moisture resistance were dramatically increased by the incorporation of only 5 wt% PI into both the graft and block copolymers. However, the introduction of an aromatic imide structure into the PA6 chain by random copolymerization is rarely reported. Different from other copolymerization strategies, random copolymerization is a one-pot method, where all the copolymerized monomers are added at the same time. These characteristics make random copolymerization particularly suitable for large-scale industrial production due to its simple process and low cost. Moreover, the properties of the random copolymer can be adjusted in a wide range. Therefore, using the random copolymerization method to synthesize the PA6 random copolymer containing an aromatic imide structure is of great significance both in theoretical research and practical applications.

In our previous work, we reported a new method for the synthesis of poly(amide imide)s (PAIs) using diimide diacid (DIDA) monomers containing different rigid aromatic groups between two imide rings to polymerize with 1,10-diaminodecane via a polycondensation process similar to that of PA66 [22]. Inspired by this synthesis, the method of PAIs was combined with our past experience in the synthesis of PA6/66 random copolymers; therefore, in this work, we first synthesized the DIDA monomer by the cyclization reaction of pyromellitic dianhydride (PMDA) and 6-aminocaproic acid and then reacted it with 1,6-hexamethylenediamine (HMDA) to form PAI salt, and finally synthesized PA6/PAI random copolymers containing an aromatic imide structure through the random copolymerization of ε-caprolactam (CL) and PAI salt. Their chemical structures and molecular weights were characterized by FTIR, ^1^H-NMR, and intrinsic viscosity measurements. Their thermal properties and crystallization behaviors were researched by TGA, DSC, WAXD, and DMTA. In addition, their rheological behaviors and mechanical and dielectric properties were also systematically investigated. Our results demonstrate how the rigid aromatic imide structure influences the properties of PA6/PAI random copolymers by comparing it with pure PA6.

## 2. Materials and Methods

### 2.1. Materials

Pyromellitic dianhydride (PMDA, 99%), 6-aminocaproic acid (98%), 1,6-hexa- methylenediamine (HMDA, 99%), N,N-dimethylformamide (DMF, 99%) and sodium acetate (NaAc, 99%) were purchased from Shanghai Aladdin Biochemical Technology Co., Ltd., Shanghai, China. Acetic anhydride (Ac2O, 99%) was purchased from Sinopharm Chemical Reagent Co., Ltd., Beijing, China. ε-Caprolactam (CL, 99%) was produced in Yueyang Chemical Fiber Co., Ltd., Yueyang, China. The materials were used directly with no further purification.

### 2.2. Synthesis of DIDA Monomer

**Scheme 1:**
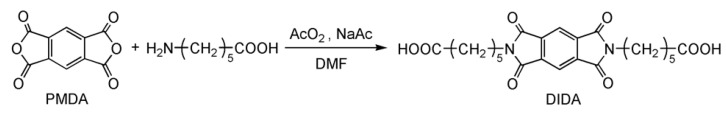
Synthetic route of DIDA monomer.

PMDA (218.0 g, 1.0 mol), 6-aminocaproic acid (275.1 g, 2.1 mol), and 1500 mL DMF were added into a dried reaction kettle and reacted at room temperature for 1 h. Then, NaAc (8.2 g, approximately equal to 0.1 mol) and Ac2O (10.2 g, approximately equal to 0.1 mol) were added to this reaction kettle. Then, the material temperature in the reaction kettle was raised to 140 °C and maintained for 6 h. Finally, the crude product was filtered and then vacuum-dried under 80 °C for 12 h. The white powder DIDA was synthesized. Yield: 386.3 g, 87%. The synthetic process of the DIDA monomer is displayed in Scheme 1.

### 2.3. Synthesis of PAI Salt

In total, 500 g of DIDA and 1000 mL of deionized water were added into a salt-forming kettle. HMDA was slowly added into this salt-forming kettle under stirring at 60 °C. When the pH value of the mixture reached 7.5–7.8, we stopped adding HMDA. The mixture stood for 4 h to make the PAI salt precipitate out before PAI salt was obtained by suction filtration and vacuum drying.

### 2.4. Synthesis of PA6/PAI Random Copolymers

**Scheme 2:**
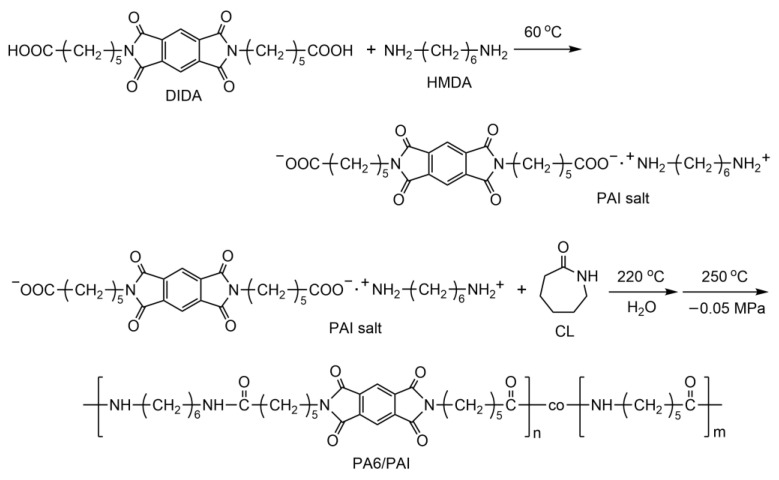
Synthetic route of PAI salt and PA6/PAI random copolymers.

PA6/PAI random copolymers containing an aromatic imide structure were polymerized through the process shown in Scheme 2. The following is a typical process of polymerization. A total of 1600 g of CL and 400 g of PAI salt were mixed with a given mass of deionized water. Then, the mixture was added into an autoclave with a volume of 5 L. The autoclave was filled with high-purity *N*_2_ until the pressure reached 0.3 MPa, and was then placed in a pumping vacuum. This process was repeated three times. The system reacted at 220 °C for 2 h; meanwhile, water vapor was released slowly to control the pressure inside the autoclave so that it did not exceed 2.5 Mpa. Then, the pressure was reduced from 2.5 Mpa to atmospheric pressure by releasing the water vapor, which was then further reduced to −0.05 Mpa by dehydrating in the vacuum. The reaction continued for a further 2 h at 250 °C and −0.05 Mpa. Finally, the polymerzation was finished, and the crude polymer containing the oligomers was extracted in boiling water for 8 h. After that, the saturated water-absorbing polymer was placed in a vacuum drying oven at 80 °C for 12 h to obtain: 

PA6/PAI-20 random copolymer. Other PA6/PAI random copolymers with a different PAI content, as described in Table 1, were synthesized by the same preparation process.

### 2.5. Characterization

Fourier transform infrared (FTIR) spectra were measured with a Nicolet IS 10 Fourier transform infrared spectrometer with attenuated total reflection (ATR) technology. ^1^H-NMR spectra were acquired on a Bruker ARX400 spectrometer at room temperature using deuterated dimethyl sulfoxide (DMSO-d_6_) or deuterated trifluoroacetic acid (CF_3_CO_2_D) as the solvent for the DIDA monomer and polymers, respectively. Tetramethylsilane (TMS) was used as the internal reference. 

Intrinsic viscosity measurements were tested with a Ubbelohde viscometer at 25 °C using 96% H_2_SO_4_ as a solvent, and the concentration of the polymer solution was 0.5 g/dL. The intrinsic viscosity [*η*] of the sample was calculated using the following Equation (1):

(1) $$\left[\eta \right]=\frac{\sqrt{2\left(\eta_{\mathrm{sp}}-ln\eta_{r}\right)}}{C}$$

where *η*_r_ = *t*/*t*_0_, *η*_sp_ = *t*/*t*_0_ − 1. *t* was the efflux time of the solution, *t*_0_ was the efflux time of the solvent, *η*_r_ was the relative viscosity, *η*_sp_ was the specific viscosity, and *C* was the concentration of the polymer solution.

Thermogravimetric analysis (TGA) was examined on a Mettler Toledo TGA/DSC1 1100SF instrument. The sample was heated from 25 °C to 700 °C at a rate of 10 °C/min in a nitrogen atmosphere. Differential scanning calorimeter (DSC) thermograms were detected using a Netzsch DSC 204 F1 at a heating and cooling rate of 10 °C/min from 25 °C to 300 °C in a nitrogen atmosphere. The crystallinity (*X*_c_) of the sample was calculated by the following Equation (2):

(2) $$X_{c}=\frac{\Delta H_{m}}{\Delta H_{m}^{0}}\;\times 100\%$$

where $\Delta H_{m}$ was the melting enthalpy of the sample obtained by integrating the melting peak of the DSC curve during the second heating process, and $\Delta H_{m}^{0}$ was the melting enthalpy of the 100% crystallized PA6 (230.1 J/g) [23].

The wide-angle X-ray diffraction (WAXD) patterns were recorded on a Bruker D8 Discover diffractometer in the 2θ range of 3–40°.

The glass transition temperature (Tg) was measured by a Q850 dynamic mechanical thermal analyzer (DMTA) at a heating rate of 5 °C/min from −70 °C to 100 °C.

Rheological properties were investigated with a Thermo Fisher Mars III rotational rheometer. The specimens with a thickness of 1 mm and a diameter of 25 mm were obtained by injection molding. Complex viscosities (η*) were tested between parallel plates at 240 °C, and angular frequencies ranged from 0.05 to 500 rad/s.

Tensile strength, Young’s modulus, and fracture elongation were detected with the method of GB/T 1040.2-2006, which was equivalent to ISO 527-2:1993. The bending strength and bending modulus were measured according to GB/T 9341-2008, which was equivalent to ISO 178:2001. The impact strength was measured according to GB/T 1043.1-2008, which is equivalent to ISO 179-1:2000. The temperature and relative humidity for the test was 23 ± 2 °C and 50 ± 10%, respectively. Each test was repeated five times, and the average value was calculated together with the standard deviation.

The dielectric constant and dielectric loss were tested using a Subo Electric QS37 high voltage bridge measurement system according to GB/T 1049-2006. The test voltage was 1000 V, the frequency was 50 Hz, and the temperature was 23 ± 2 °C.

## 3. Results and Discussion

### 3.1. Structure Characterization of DIDA Monomer

**Figure 1:**
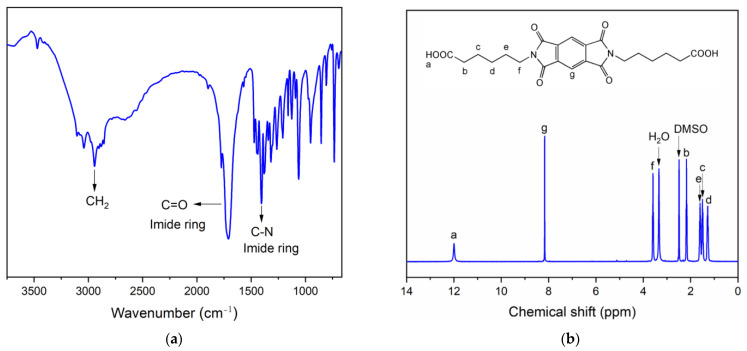
FTIR spectrum (**a**) and ^1^H-NMR spectrum (**b**) of DIDA monomer.

As shown in Scheme 1, the DIDA monomer was synthesized by the dehydration cyclization of PMDA and 6-aminocaproic acid, and its structure was characterized by FTIR and ^1^H-NMR. Figure 1a shows the FTIR spectrum of the DIDA monomer. It can be observed that the characteristic absorption peaks appeared at 1712 cm^−1^ (C=O, imide ring) and 1398 cm^−1^ (C–N stretching, imide ring), which indicated the successful formation of the imide ring [24,25]. The ^1^H-NMR spectrum of the DIDA monomer in DMSO-d6 is shown in Figure 1b. The characteristic resonance peaks at 3.48–3.68 ppm were the proton peaks of the methylene connected with the imide group (denoted f), which suggested that the dehydration cyclization reaction was carried out, as described in Scheme 1.

### 3.2. Structure Characterization of PA6/PAI Random Copolymers

PA6/PAI random copolymers with a different PAI content were obtained by the random copolymerization of CL and PAI salt, and their polymerization process was similar to that of PA6/66 random copolymers. Deionized water was used as the reaction medium because it is more environmentally friendly and cheaper than organic solvents. The system reacted at 220 °C for 2 h, where CL underwent a hydrolytic ring-opening reaction to form 6-aminocaproic acid, and 6-aminocaproic acid and PAI salt was converted to the oligomer at 220 °C and 2.5 MPa. The degree of polymerization of the oligomer gradually increased with the water vapor. In order to further improve the degree of polymerization, the reaction was continued for a further 2 h at 250 °C and −0.05 MPa. After the reaction ended, PA6/PAI random copolymers were obtained.

**Figure 2:**
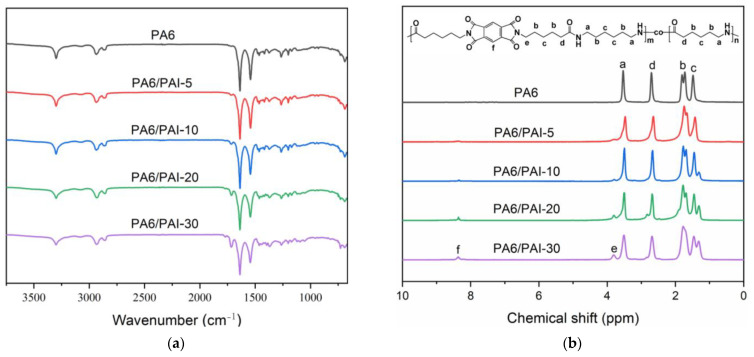
FTIR spectra (**a**) and ^1^H-NMR spectra (**b**) of PA6/PAI random copolymers.

The chemical structures of PA6/PAI random copolymers were verified by FTIR and ^1^H-NMR. Figure 2a shows the FTIR spectra of PA6/PAI random copolymers. The spectral features at 1640 cm^−1^ (C=O, amide) and 1542 cm^−1^ (N-H, C-N, amide) directly verified the formation of an amide bond. Furthermore, the characteristic absorption peak of the imide ring of PA6/PAI random copolymers could still be observed at 1712 cm^−1^, and the intensity of this peak increased with the increasing PAI content.

Figure 2b shows the 1H-NMR spectra of PA6/PAI random copolymers in CF3CO2D. The methylene proton peaks connected with the amide group (denoted a) and connected with the imide group (denoted e), respectively, appeared at 3.36–3.68 ppm and 3.70–3.90 ppm, which demonstrated the existence of amide bonds and imide rings in PA6/PAI randomcopolymers. Furthermore, the chemical shifts of other protons in ^1^H-NMR spectra were also consistent with the designed PA6/PAI random copolymers, and the strength of the methylene proton peak (denoted e) when connected with imide group and benzene proton peak (denoted f) increased with the increase in the PAI content. Therefore, it could be inferred by the FTIR and ^1^H-NMR spectra that the chemical structures of PA6/PAI random copolymers were as expected.

The viscosity-average molecular weights ($\bar{M_{\eta }}$) of PA6/PAI random copolymers are usually calculated by intrinsic viscosities [*η*] according to the Mark–Houwink equation, $\left[\eta \right]=K{\bar{M_{\eta }}}^{\alpha }$, where *K* and *α* are constants that only depend on the properties of the polymer solution. However, PA6/PAI random copolymers are new polymers, and their values of K and α are not available in the literature. Therefore, we could only estimate the $\bar{M_{\eta }}$ of the PA6/PAI random copolymers based on the *K* and *α* of PA6, and its values were 2.26 × 10^−4^ and 0.82, respectively [26,27,28]. As shown in Table 1, the measured [*η*] values of the PA6/PAI random copolymers ranged from 0.82 to 0.95 dL/g, and the calculated $\bar{M_{\eta }}$ values were all greater than 21,000, which indicated that the PA6/PAI random copolymers with high molecular weights could be successfully synthesized by the random copolymerization method.

Although the molecular weights of PA6/PAI random copolymers were slightly different, this had little effect on the performance of the materials. Therefore, in the following work, we mainly focused on the influence of the PAI content and on the properties of PA6/PAI random copolymers.

### 3.3. Thermal Properties and Crystallization Behaviors

**Figure 3:**
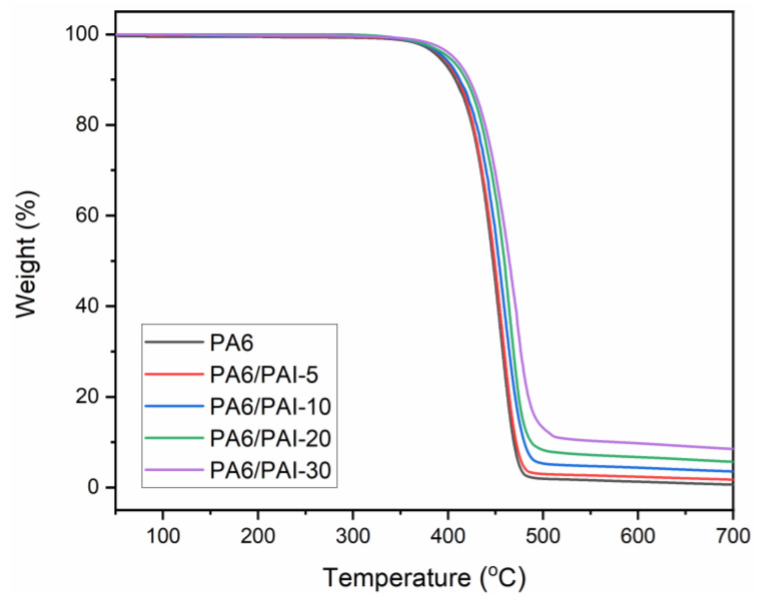
TGA curves of PA6/PAI random copolymers.

The thermal stabilities of PA6/PAI random copolymers were investigated by TGA. Figure 3 demonstrates the TGA graphs of PA6/PAI random copolymers in the *N*_2_ atmosphere, and the initial thermal decomposition temperatures (*T*_d_) at 5% weight loss and char yields at 700 °C are listed in Table 2. Compared with pure PA6, PA6/PAI random copolymers exhibited better thermal stabilities, and the *T*_d_ value and char yield of the PA6/PAI random copolymer increased obviously with the increase in the PAI content. The results indicate that the introduction of an aromatic imide structure into the PA6 chain improved its thermal stability.

**Figure 4:**
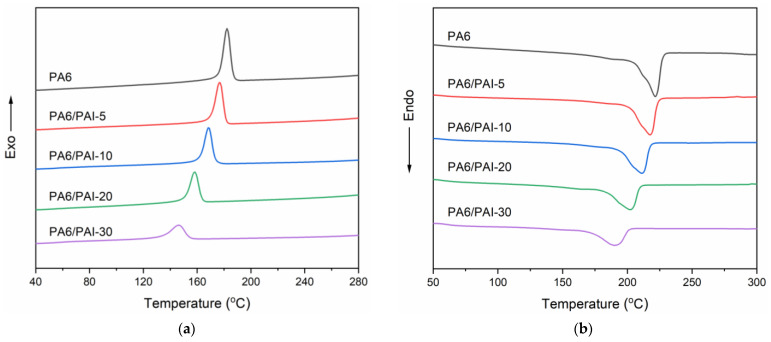
DSC curves of PA6/PAI random copolymers during the first cooling (**a**) and the subsequent heating (**b**) at a rate of 10 °C min^−1^.

The DSC curves of PA6/PAI random copolymers during the first cooling and subsequent heating process are shown in Figure 4, and the characteristic parameters are listed in Table 2. The content of PAI had a great influence on the melting and crystallization behaviors of PA6/PAI random copolymers. As shown in Figure 4, the crystallization temperature (*T*_c_) and melting point (*T*_m_) of the PA6/PAI random copolymer gradually decreased with the increase in the PAI content. The *T*_c_ and *T*_m_ of PA6 were 182.2 °C and 221.8 °C, while the *T*_c_ and *T*_m_ of PA6/PAI-30 were 145.9 °C and 190.5 °C. These results suggest that the more aromatic imide groups that were introduced into the PA6 chain, the more irregular the structure of the copolymer was, the ordered arrangement of the chain segments was more difficult, and fewer crystals were formed. Moreover, with the increase in the PAI content, the melting peak of the PA6/PAI random copolymer became ever lower and wider. 

The melting enthalpy (Δ*H*_m_) obtained by integrating the melting peak of the DSC curve during the second heating process is listed in Table 2. With the aid of the melting enthalpy of 100% crystallized PA6 (230.1 J/g), the crystallinity (*X*_c_) was calculated by Equation (2). As shown in Table 2, the *X*_c_ of the PA6/PAI random copolymer was obviously lower than that of pure PA6, and the *X*_c_ of PA6/PAI random copolymer decreased with the increasing content of PAI, especially in the region with a high PAI content. The variation trend of *X*_c_ was the same as that of *T*_c_ and *T*_m_. Therefore, the introduction of an aromatic imide structure into the PA6 chain limited the flexibility and mobility of the chain segment and reduced the crystallization ability of the PA6/PAI random copolymer.

**Figure 5:**
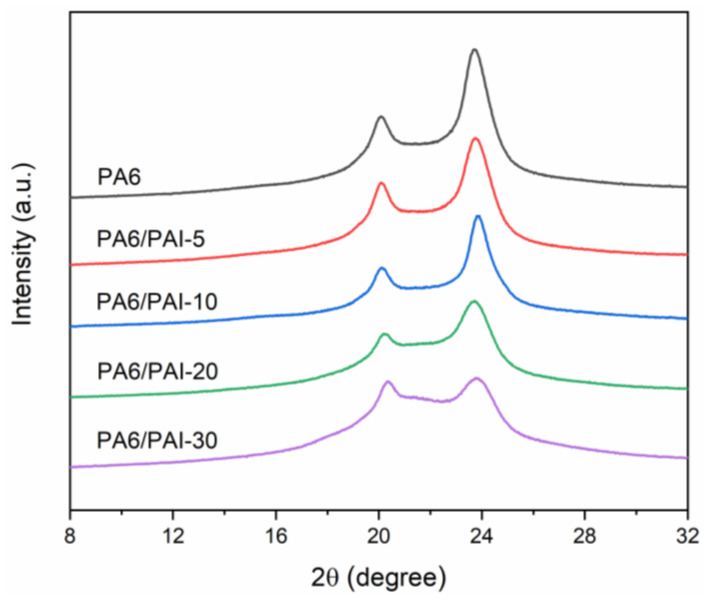
WAXD patterns of PA6/PAI random copolymers.

The crystal structure of PA6/PAI random copolymers was researched by WAXD, and these curves are demonstrated in Figure 5. Pure PA6 had two diffraction peaks at 2θ = 20.08° and 23.72°, which showed that the crystal structure belonged to *α*-form. Compared with pure PA6, the positions of these two diffraction peaks of PA6/PAI random copolymer were: 

It was almost crystal in form but led to a decrease in crystallinity. The introduction was unchanged, but their intensities decreased significantly with the increasing PAI content. The results indicate that the introduction of the aromatic imide structure did change the aromatic imide structure breaks of the original regular chain structure of PA6, making it difficult to arrange the chain segments into the lattice regularly. The results of WAXD were consistent with those of DSC.

**Figure 6:**
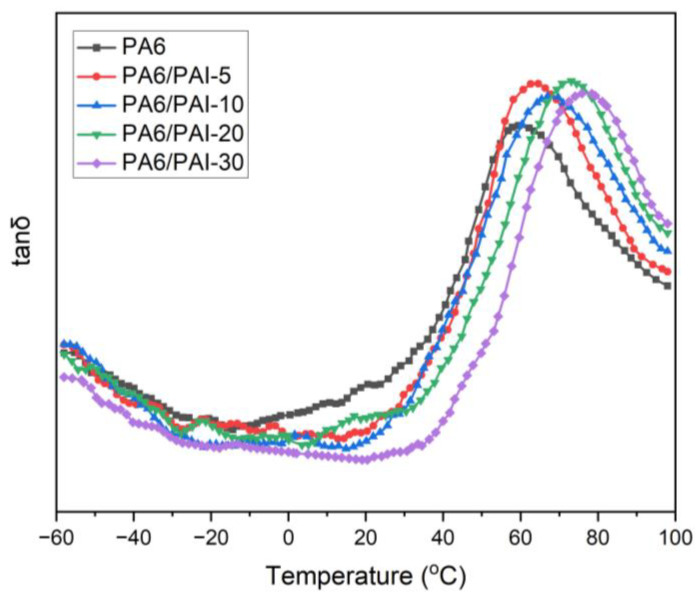
The curves of tan δ relating to the temperature for PA6/PAI random copolymers.

The glass transition temperature (*T*_g_) of PA6/PAI random copolymers was measured by DMTA. Figure 6 shows the curves of tan δ relating to the temperature for PA6/PAI random copolymers, and the *T*_g_ values are listed in Table 2. Note that the *T*_g_ values of PA6/PAI random copolymers were obviously higher than that of PA6, indicating the improved rigidity of the chain segment due to the introduction of an aromatic imide structure. The higher the PAI content, the higher the *T*_g_ value of PA6/PAI random copolymers. The change in the chain segment rigidity had a great influence on the rheological properties, mechanical properties, and dielectric properties of PA6/PAI random copolymers, and the latter two properties were also affected by the crystallinity.

### 3.4. Rheological Properties

**Figure 7:**
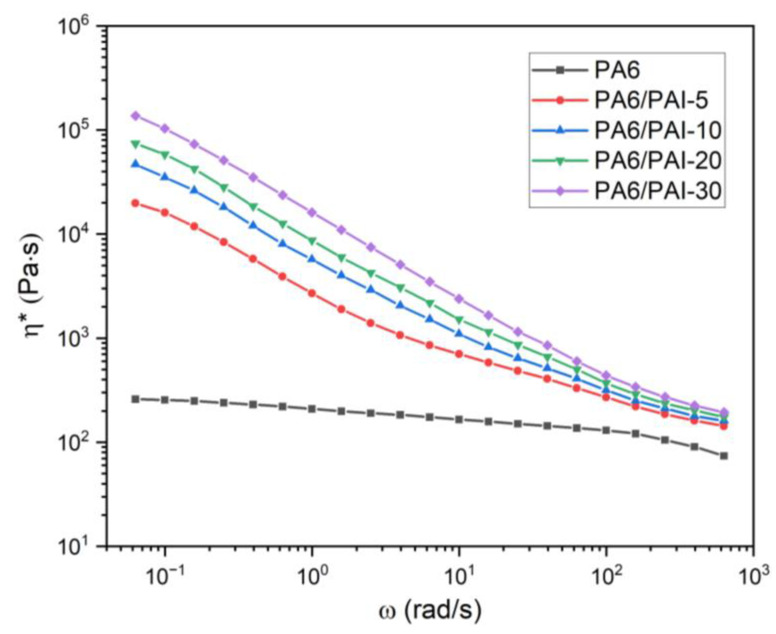
Complex viscosities (η*) of PA6/PAI random copolymers.

Rheological properties are important for polymer processing and are usually researched by a rotational rheometer. Figure 7 shows the complex viscosity (η*) of PA6/PAI random copolymers with different PAI content. As the frequency increased, all the samples exhibited the shear thinning behavior of the pseudoplastic fluid, which could be attributed to the molecular chains’ orientation along the flow direction, which was untangled at a high shear frequency [29,30]. Compared with PA6, the η* of PA6/PAI random copolymer increased significantly due to the introduction of the rigid aromatic imide structure. Moreover, the content of PAI had a great effect on the η* of the PA6/PAI random copolymer. With the increase in the PAI content, the η* of the PA6/PAI random copolymer increased. The results indicate that the more rigid the aromatic imide groups in the copolymer, the more difficult the movement of the copolymer chain was [31]. Despite the increased viscosities of PA6/PAI random copolymers, they could be processed at lower temperatures due to their low melting points.

### 3.5. Mechanical Properties

**Figure 8:**
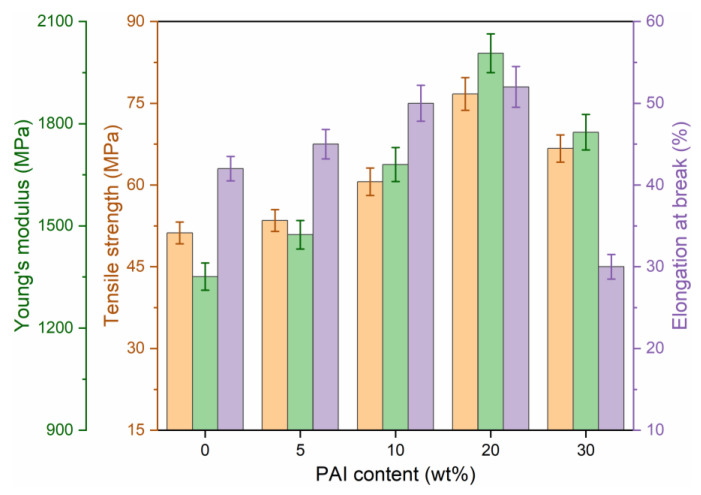
Tensile properties of PA6/PAI random copolymers.

**Figure 9:**
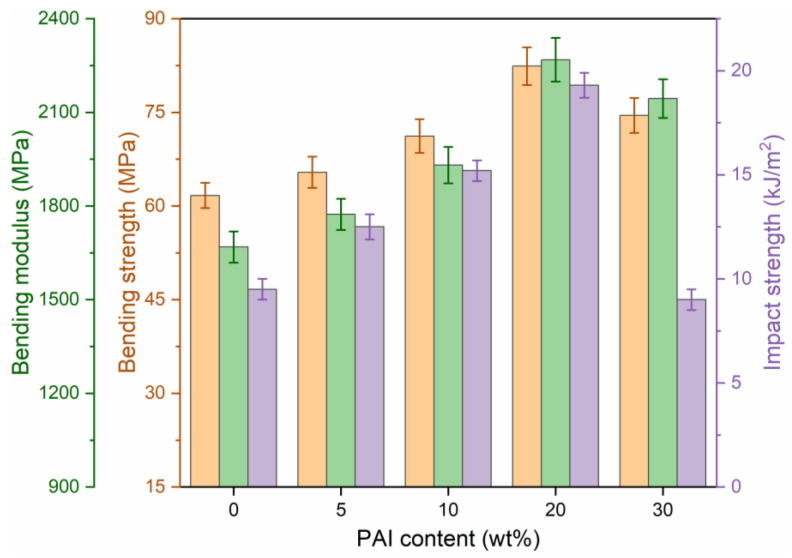
Bending and impact properties of PA6/PAI random copolymers.

The mechanical properties of PA6/PAI random copolymers with a different proportion of PAI are demonstrated in Figure 8 and Figure 9. When the content of PAI was 20 wt% or less, the tensile strength, Young’s modulus, bending strength, and bending modulus of the PA6/PAI random copolymer obviously increased with the increasing PAI content, and these mechanical properties were significantly higher than those of PA6, which indicated that the introduction of an aromatic imide structural unit improved the rigidity of the chain segment. When the content of PAI reached 30 wt%, these mechanical properties appeared to decline because excessive aromatic imide groups seriously destroyed the crystallization of the PA6 chain segment. Meanwhile, the impact strength and fracture elongation of the PA6/PAI random copolymer also increased first and then decreased and reached the maximum when the PAI content was 20 wt%. A few aromatic imide groups acted as a plasticizer in PA6, improving the plasticity of the copolymer. However, excess aromatic imide groups can also greatly reduce the slip ability between molecular chains, and the toughness of PA6/PAI-30 can deteriorate significantly. Therefore, PA6/PAI-20 had the best mechanical properties.

### 3.6. Dielectric Properties

**Figure 10:**
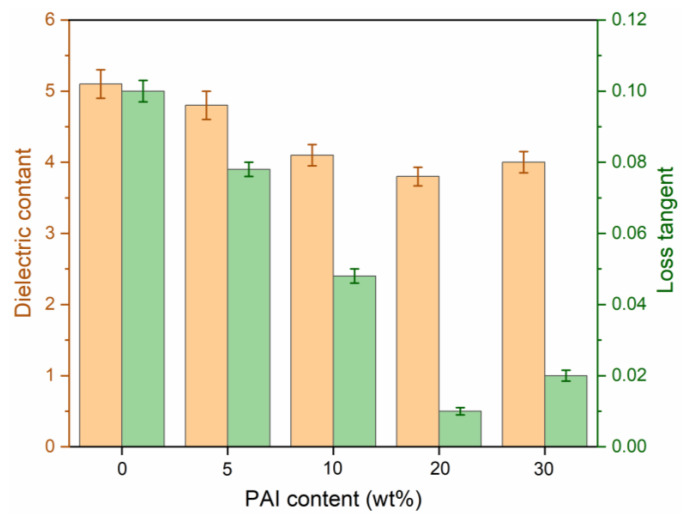
Dielectric constants and loss tangents of PA6/PAI random copolymers.

The dielectric properties of PA6/PAI random copolymers were evaluated by the dielectric constant and dielectric loss, as shown in Figure 10. The changes in the dielectric constant and dielectric of the PA6/PAI random copolymer could mainly be related to the number of orientable dipoles and the ability of dipole motion [32]. With the increase in the PAI content, the dielectric constant and dielectric loss of the PA6/PAI random copolymer decreased first and then increased. When the content of PAI was in the range of 0–20 wt%, the increasing aromatic imide groups improved the rigidity of the chain segment and impeded the motion of the dipole, which was not conducive to the polarization of the dipole but led to the reduction in the dielectric constant and dielectric loss. The dielectric constant and dielectric loss of PA6/PAI-20 markedly decreased to 3.85 and 0.01 from 5.12 and 0.10 for PA6, respectively. After that, the dielectric constant and dielectric loss of the PA6/PAI random copolymer increased with the further increase in the PAI content. This was because the introduction of excessive aromatic imide groups seriously destroyed the crystallization of the copolymer and increased the number of orientable dipoles, thus increasing the dielectric constant and dielectric loss.

## 4. Conclusions

In this work, the DIDA monomer was obtained by the dehydration cyclization of PMDA and 6-aminocaproic acid, which was then reacted with HMDA to form PAI salt. PA6/PAI random copolymers with a different PAI content were successfully synthesized via the random copolymerization of CL and AI salt, and their thermal properties, crystallization, rheological behaviors, and mechanical and dielectric properties were systematically investigated. The results indicate that PA6/PAI random copolymers containing aromatic imide structure exhibit excellent thermal stabilities, and the *T*_d_ value and char yield increase obviously with the increase in the PAI content. The introduction of the aromatic imide structure did not change the crystal form but led to a decrease in crystallinity. As the proportion of PAI increases, the *T*_c_ and *T*_m_ values of the PA6/PAI random copolymer gradually decrease, but its *T*_g_ value increased obviously. Compared with pure PA6, the η* values of PA6/PAI random copolymers were higher, but they still had good processabilities because of their lower *T*_m_ values. The introduction of an aromatic imide structural unit into the PA6 chain had a great influence on their mechanical and dielectric properties. When the PAI content was 20 wt%, the copolymer PA6/PAI-20 not only had high strength, modulus, and toughness but also demonstrated a low dielectric constant and dielectric loss. These results indicate that this method was feasible for designing and synthesizing new PA6 copolymers with high thermal stabilities and excellent mechanical and dielectric properties.

## Figures and Tables

**Table 1:** The synthesis of PA6/PAI random copolymers with different PAI content.

Sample	CL (g)	PAI Salt (g)	[*η*] ^a^ (dL/g)	$\bar{M_{\eta }}{\;}^{a}\;(\times 10^{4})$
PA6	2000	0	1.02	2.86
PA6/PAI-5	1900	100	0.95	2.62
PA6/PAI-10	1800	200	0.82	2.19
PA6/PAI-20	1600	400	0.86	2.32
PA6/PAI-30	1400	600	0.91	2.49

^a^ [*η*] was the intrinsic viscosity, and $\bar{M_{\eta }}$ was the viscosity-average molecular weights.

**Table 2:** Thermal properties of PA6/PAI random copolymers.

Sample	*T*_d_ ^a^ (°C)	Char Yield ^a^ (%)	*T*_c_ ^b^ (°C)	*T*_m_ ^c^ (°C)	Δ*H*_m_ ^c^ (J/g)	*X*_c_ ^d^ (%)	*T*_g_ ^e^ (°C)
PA6	392.1	0.6	182.2	221.8	58.0	25.2	60.5
PA6/PAI-5	393.8	1.7	176.4	217.7	55.7	24.2	63.8
PA6/PAI-10	395.7	3.5	168.2	211.1	54.1	23.5	67.4
PA6/PAI-20	400.2	5.6	158.3	202.1	49.8	21.6	73.1
PA6/PAI-30	405.4	8.4	145.9	190.5	36.5	15.9	77.2

^a^ The initial thermal decomposition temperatures *T*_d_ at 5% weight loss and char yields at 700 °C were measured by TGA at a rate of 10 °C/min under *N*_2_. ^b^ The crystallization temperature *T*_c_ was obtained by DSC during the cooling process. ^c^ The melting point *T*_m_ and the melting enthalpy Δ*H*_m_ were obtained by DSC during the second heating process. ^d^ *X*_c_ = Δ*H*_m_/$\Delta H_{m\;}^{0}$, $\Delta H_{m}^{0}$ = 230.1 J/g. ^e^ The glass transition temperature (*T*_g_) was obtained by DMTA at a heating rate of 5 °C/min from −70 °C to 100 °C.

## Data Availability

Not applicable.
